# Predicting Δ‑9-Tetrahydrocannabinol-Induced Psychoactive and Cognitive Effects: A PBPK–PD Approach to Quantifying Feeling High and Reduced Alertness

**DOI:** 10.1021/acschemneuro.5c00417

**Published:** 2025-07-22

**Authors:** Lixuan Qian, Zhu Zhou

**Affiliations:** Department of Chemistry, York College, 2009 City University of New York, Jamaica, New York 11451, United States

**Keywords:** Δ-9-tetrahydrocannabinol, psychoactive, feeling high, alertness, PBPK−PD, pharmacodynamics

## Abstract

The increasing use of cannabis for medicinal and recreational purposes highlights the need to understand its psychoactive effects. Δ-9-tetrahydrocannabinol (THC), the primary psychoactive cannabinoid, is responsible for feeling high and reduced alertness after cannabis use. This study aimed to develop and verify physiologically based pharmacokinetic–pharmacodynamic (PBPK–PD) models to quantify the effects of THC and its active metabolite, 11-hydroxy-THC, on feeling high and reduction in alertness in healthy adults. The models were developed using Simcyp, based on our previously verified THC PBPK model. A direct response model with a maximum effect (*E*_max_) function driven by the brain concentrations and an effect compartment was used to describe visual analogue scale (VAS) scores for feeling high after intravenous, oral, and inhaled THC administration. An indirect response model with an *E*_max_ function driven by the brain concentrations was used to describe the reduction in VAS alertness scores after inhaled THC. Our models accurately captured the dose–response relationships for THC doses ranging from 2 to 86 mg for feeling high, and 2 to 69.4 mg for alertness reduction. The verified PBPK–PD model provides a robust tool for predicting the psychoactive and cognitive effects of THC, enabling improved assessment of cannabis-induced responses across diverse populations.

## Graphical abstract

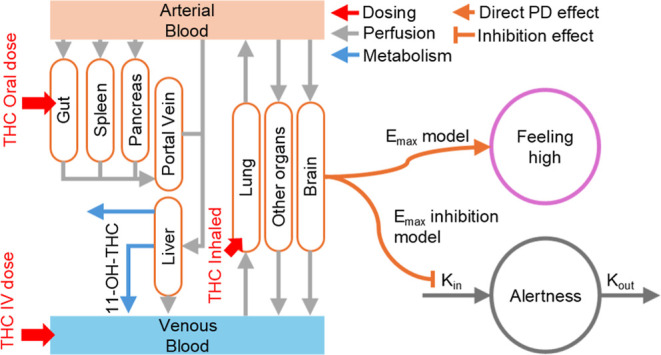

## 1 Introduction

Cannabis use in the United States (U.S.) has increased dramatically over the past decade.^1^ In 2022, an estimated 62 million individuals in the U.S. aged 12 and older (22% of the population) reported using cannabis in the past year.^2^ Concurrently, the primary psychoactive component of cannabis, Δ-9-tetrahydrocannabinol (THC), has become more potent. While some U.S. states have legalized cannabis products with THC concentrations ≤0.3%,^3^ the average THC concentration in illegal cannabis samples increased from approximately 4% in 1995 to 13% in 2022.^4,5^ As cannabis use becomes more widespread and THC potency increases, significant concerns grow regarding THC’s psychoactive and cognitive effects, such as feeling high and reduced alertness.

THC has high permeability (Log *P*: 6.97) and low aqueous solubility (2–10 μg/mL),^6,7^ leading to low and variable bioavailability when administered orally (4–20%).^1^ Due to its lipophilic nature, THC has a large volume of distribution and accumulates extensively in brain tissue, where its concentrations are 2–5 fold higher than in plasma.^8^ After administration, THC mainly binds to cannabinoid receptor 1 (CB1), with its psychoactive and cognitive effects primarily mediated through CB1 activation in the brain.^9^ The CB1, the most abundant G protein-coupled receptor in the brain, is expressed differentially across brain regions with particularly high densities in the cerebral cortex (especially prefrontal regions and hippocampus), amygdala, cerebellum, and basal ganglia.^10,11^

The subjective experience of feeling high is the primary motivation for recreational cannabis use and contributes to its abuse potential.^12–14^ The term feeling high is an underdefined concept that is often used to describe the hyper-sensory experience after cannabis use.^15^ Research has demonstrated that a greater feeling high is significantly associated with higher THC doses compared to other factors.^16^ To quantify this subjective state, researchers commonly use the visual analogue scale (VAS) to determine the degree of feeling high, typically using a 100 mm line where participants mark their current state between “not high at all” (0 mm) and “extremely high” (100 mm). Acute THC exposure has been shown to alter baseline brain perfusion and neural activity, particularly in brain areas involved in cognitive processing, emotional regulation, and interoceptive awareness.^12,17^ CB1 activation is known to mediate this response, although the precise signal transduction pathways linking receptor engagement to subjective effects remain incompletely characterized.

Another key central nervous system (CNS) effect of THC is reduced alertness, which has significant public health implications.^18^ Alertness, defined as a behavioral and physiological state of being able to respond appropriately to stimuli, is critical for cognitive function, occupational performance, and overall quality of life.^19^ Reduced alertness has been linked to decreased workplace productivity, absenteeism, and a higher risk of work-related accidents.^19^ It is often measured subjectively using the VAS, where it is considered the opposite of sleepiness.^20^ The participants mark their current state between “drowsy” (0 mm) and “alert” (100 mm), and functionally as the manifestation of the alerting network. Clinical and animal studies have shown that norepinephrine (NE), dopamine (DA), and 5-hydroxytryptamine (5-HT) are key neurotransmitters that modulate alertness.^21–24^ Noradrenergic modulation can influence the efficiency of the alerting network, while dopaminergic modulation can influence orienting and executive control of attention.^25^ NE plays a critical role in maintaining alertness through continuous activation of the locus coeruleus–norepinephrine (LC–NE) system, and the NE concentration in the LC is reported to be decreased after THC administration.^24,26^ CB1 is abundantly expressed in dopaminergic neurons, where they modulate DA transmission through a retrograde feedback system. A decrease in D2/D3 receptor availability in the ventral striatum has been associated with reduced alertness.^27^ Additionally, 5-HT receptors regulate the release of both NE and DA.^28,29^ The tonic aspect of alertness is also maintained by an intense release of 5-HT induced by persistent activation of either 5-HT neurons or local 5-HT axon terminals.^30^ In an *in vitro* study, THC administration rapidly and thoroughly inhibited the 5-HT activation and reached 90% of maximal 5-HT receptor inhibition in less than 1 min.^31^ While the details of these mechanisms require further study, existing evidence strongly suggests that THC-induced alterations in neurotransmitter signaling contribute to reduced alertness.

Understanding how THC modulates these psychoactive and cognitive responses is critical. Many clinical trials have provided valuable insights, and quantitative modeling techniques, such as population pharmacokinetics/pharmacodynamics (popPK/PD), have been used to examine the relationship between THC dose and VAS measure of feeling high and alertness.^32–34^ However, these models have notable limitations. They do not account for the synergistic effects of 11-hydroxy-THC (11-OH-THC), a primary active metabolite of THC, and only consider plasma THC concentrations. Additionally, extrapolation for the popPK/PD model is limited by the characteristics of the studied populations, making it challenging to predict feeling high and its effect on alertness across various dosages, routes of administration, and demographic groups.

The physiologically based pharmacokinetic (PBPK) model offers a more mechanistic approach by incorporating the physicochemical properties of the drug and physiological and biological knowledge at the organism level.^35^ PBPK models can provide a more accurate description of drug exposure in various organs, including the brain. By leveraging organ-level concentration predictions, physiologically based pharmacokinetic–pharmacodynamics (PBPK–PD) models can establish direct relationships between THC exposure in the brain and its effects. Unlike empirical popPK/PD models, PBPK–PD allows for extrapolation across diverse populations and administration routes.^35^ In our prior study, organ concentrations have been shown to predict pharmacodynamic (PD) responses more accurately than plasma concentrations.^36^ Additionally, unlike previous popPK/PD models that focus solely on THC, PBPK–PD models can incorporate the effects of 11-OH-THC, enabling a more comprehensive assessment of exposure–response relationships related to both feeling high and alertness reduction.

This research aims to address existing gaps by developing and verifying a PBPK–PD model to predict THC- and 11-OH-THC-induced changes in feeling high and alertness. By incorporating organ-level concentration predictions and accounting for key metabolites, this model aims to overcome existing limitations in PK/PD approaches, providing a more physiologically relevant framework for assessing cannabis-related CNS effects.

## 2 Results and Discussion

Feeling high and reduced alertness are among the most studied psychoactive and cognitive effects of THC, both driven by THC-activated CB1 in the brain. While these effects are well-documented, the dose–exposure–response relationship has only been described through limited popPK analyses. Previous popPK analyses have been limited by their reliance on plasma concentrations rather than brain concentrations, inability to account for active metabolites, and restricted application to specific administration routes and populations studied. This significantly limits the ability to extrapolate these effects to diverse populations and dosing regimens and, critically, to directly correlate exposure with the neurochemical events at the target site. This study addresses these gaps by developing and verifying PBPK–PD models for VAS “feeling high” and VAS “alertness” using THC and its key metabolite (11-OH-THC) concentrations predicted specifically in the brain. By incorporating the physicochemical properties of the drug and physiological and biological knowledge, our model offers a more detailed description of drug exposure at the site of action, which is the brain. This is crucial for accurately predicting the dose–exposure–response relationship for THC’s psychoactive and cognitive effects. To the best of our knowledge, this is the first modeling study to investigate the effects of THC on VAS “feeling high” and VAS “alertness” using THC concentrations at the site of action. The ability to simulate target tissue concentrations and link them to PD effects represents a significant step forward. Our PBPK–PD models accurately predicted both VAS “feeling high” and VAS “alertness”, offering a significant advancement in understanding and quantifying THC’s effects.

### 2.1 PBPK–PD Model for VAS “Feeling High”

The PD model for VAS “feeling high” was developed using a direct effect model in Simcyp, coded using the custom Lua Models within the software. The structural model for VAS “feeling high” was modified based on Strougo et al.^33^ In Strougo’s model, feeling high was related to THC concentration in the effect compartment associated with plasma.^33^ In our model, the increase in the feeling high was driven by the total THC and 11-OH-THC concentration in the effect compartment associated with the brain. This direct linkage of brain target site concentrations to the subjective effect is a key refinement. We assume that the maximum effect on VAS “feeling high” (*E*_max,high_) and concentration required to obtain 50% of the maximum change of VAS “feeling high” (EC_50,high_) are the same between THC and 11-OH-THC, and the feeling high models after THC administration were assumed to be driven by the sum of total THC and 11-OH-THC concentrations in their effect compartment. The assumptions were based on the finding that the THC and 11-OH-THC share a similar binding affinity to CB1 and exhibit comparable potency.^37,38^ All the absorption parameters for intravenous (IV), oral, and inhaled routes of administration were from our previously published studies (Table 1), and the predicted versus observed concentration–time profiles for inhaled THC were shown in our prior publications.^7,36^

**Table 1:** Final Input Parameters for the Δ-9-Tetrahydrocannabinol (THC) Absorption Model, and THC and 11-Hydroxy-THC (11-OH-THC) Pharmacodynamics Model for VAS “Feeling High” and VAS “Alertness”^a^

	THC		11-OH-THC	
parameter	value	reference	value	reference
Absorption				
model type	first-order			
*f*_a_	0.45	Qian 2025a		
*k*_a_ (h^–1^)	0.7	Qian 2025a		
Lung *f*_a_	0.22^b^, 0.025^b^, 0.05^b^, 0.4^b^, 0.6^c^, 0.9^b^	Qian 2025a;^7^ Qian 2025b^36^		
Lung *k*_a_ (h^–1^)	12^b^, 200^c^	Qian 2025a;^7^ Qian 2025b^36^		
VAS “Feeling High” PD Model				
model type	custom Lua model		custom Lua model	
*k*_e0_ (/h)	2	optimized	2	same as THC
*E*_max,high_ (%)	80	optimized	80	same as THC
EC_50,high_ (μM)	0.21	optimized	0.21	same as THC
Hill1	1.8	optimized	1.8	same as THC
CV *E*_max,high_ (%)	34	Strougo 2008 ^33^	34	same as THC
CV EC_50,high_ (%)	70	reduced from 126%^33^ to ensure positive values during simulations	70	same as THC
VAS “Alertness” PD Model				
model type	custom Lua model		custom Lua model	
*k*_out_ (/h)	1	optimized		
*E*_max,alertness_ (%)	90	optimized	90	same as THC
EC_50,alertness_ (μM)	0.23	optimized	0.23	same as THC
Hill2	1.9	optimized	1.9	same as THC
CV *E*_max,alertness_ (%)	30	default	30	same as THC
CV EC_50,alertness_ (%)	30	default	30	same as THC

a *f*_a_, fraction absorbed from dosage form; *k*_a_, first-order absorption rate constant; Lung *f*_a_, fraction of drug absorbed from the lung; *k*_e0_, transport rate constants for the effect compartment; *E*_max,high_, maximum effect on VAS “feeling high”; EC_50,high_, concentration required to obtain 50% of the maximum change of VAS “feeling high”; Hill1, power parameter for “feeling high” model; CV, coefficient of variation; *k*_out_, first-order decline constant of VAS “alertness”; *E*_max,alertness_ maximum effect on VAS “alertness”; EC_50,alertness_, concentration required to obtain 50% of the maximum change of VAS “alertness”; Hill2, power parameter for “alertness” model.

b Parameter value from Qian 2025a.^7^

c Parameter value from Qian 2025b.^36^

Our model accurately captured the peak VAS “feeling high” scores across a wide range of doses (2–86 mg) and administration routes. Figure 1 compares the model-predicted peak and observed VAS “feeling high” scores.^33,39–45^ *E*_max,high_, EC_50,high_, and transport rate constants for the effect compartment (*k*_e0_) were optimized for this model and are listed in Table 1. The highest (by D’Souza et al.^39^) and lowest (by Strougo et al.^33^) systemic exposures of THC, considering the fraction of THC absorbed, were used in the parameter optimization. The PBPK–PD model successfully captured the VAS “feeling high” score changes after IV, oral, and inhaled THC administration. In all simulated dose regimens, 94% of the predicted peak VAS “feeling high” scores were within 2-fold of the observed peak, with the ratio of predicted to observed mean peak VAS “feeling high” score (*R*_max,feeling high_) ranging from 0.46 to 1.44 (Figure 1, Table S1). The peak VAS “feeling high” score for 29.8 mg of inhaled THC is observed to be higher than that for 86 mg.^43,45^ This can be attributed to differences in the fraction of drug absorbed by the lung (Lung *f*_a_), a factor significantly influenced by the specific inhalation methodologies used across studies. The strong agreement between predicted and observed VAS “feeling high” scores across multiple administration routes and dosing regimens demonstrates the model’s robustness and supports its potential application for predicting subjective psychoactive effects in scenarios where clinical data are unavailable.

**Figure 1:**
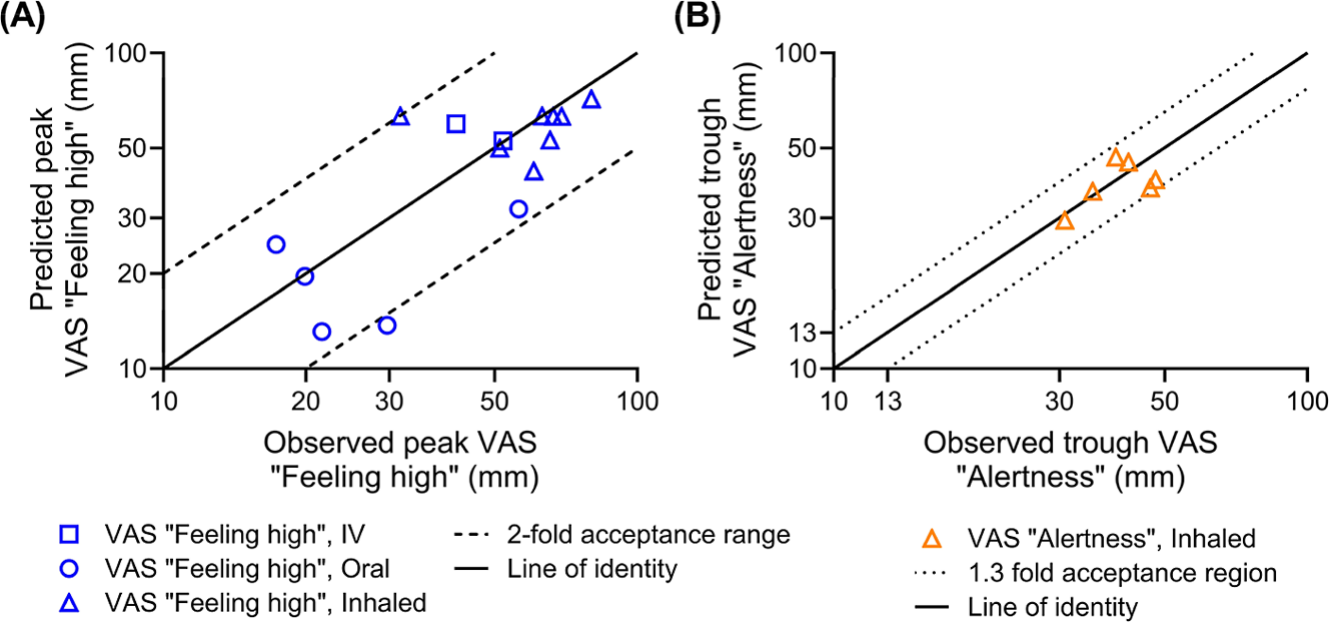
Observed vs PBPK–PD model-predicted (A) peak VAS “feeling high” and (B) trough VAS “alertness” following THC administration to healthy adults by multiple routes of administration.

Furthermore, 91% of the observed VAS “feeling high” scores fell within the fifth to 95th percentile of the predictions after THC administration (Figure 2). In Schoedel’s oral THC study with two dose regimens, a distinct pattern existed, with two peaks in VAS “feeling high” scores, one before and one after 3 h postdose.^42^ This study had a 12 h sampling window, but the timing of meal intake was not reported. Another 12 h oral study, by Wachtel and de Wit, reported a meal intake after 3 h post THC dose, and a similar two-peak pattern was observed. In Wachtel’s study, meal intake could explain the two peaks, as it can increase oral availability and delay the absorption of THC.^40^ Therefore, in Wachtel’s study, VAS scores after 3 h postdose were not presented in Figure 2. Considering the similar trial design, the meal intake could explain the two peaks phenomena in Schoedel’s study.^42^ This explanation is further supported by other THC clinical trials which demonstrated similar second peak trends around the meal times postdose, where high-fat food significantly increased the time to peak plasma concentration (*t*_max_) and exposure for THC.^46,47^ Another possible explanation for the second peak could be enterohepatic circulation (EHC), where the metabolite of the THC in bile is released to the intestinal track after a meal, metabolized back into the parent drug by intestinal bacteria, and then reabsorbed.^48^ However, only 10–15% of the THC metabolites were reported to be involved in the THC EHC.^49^ Since the percentage of the THC involved in EHC is low and the EHC of THC was not reported in other administration routes, especially IV, the higher second peak of “feeling high” is unlikely to be triggered by the EHC of THC.

**Figure 2:**
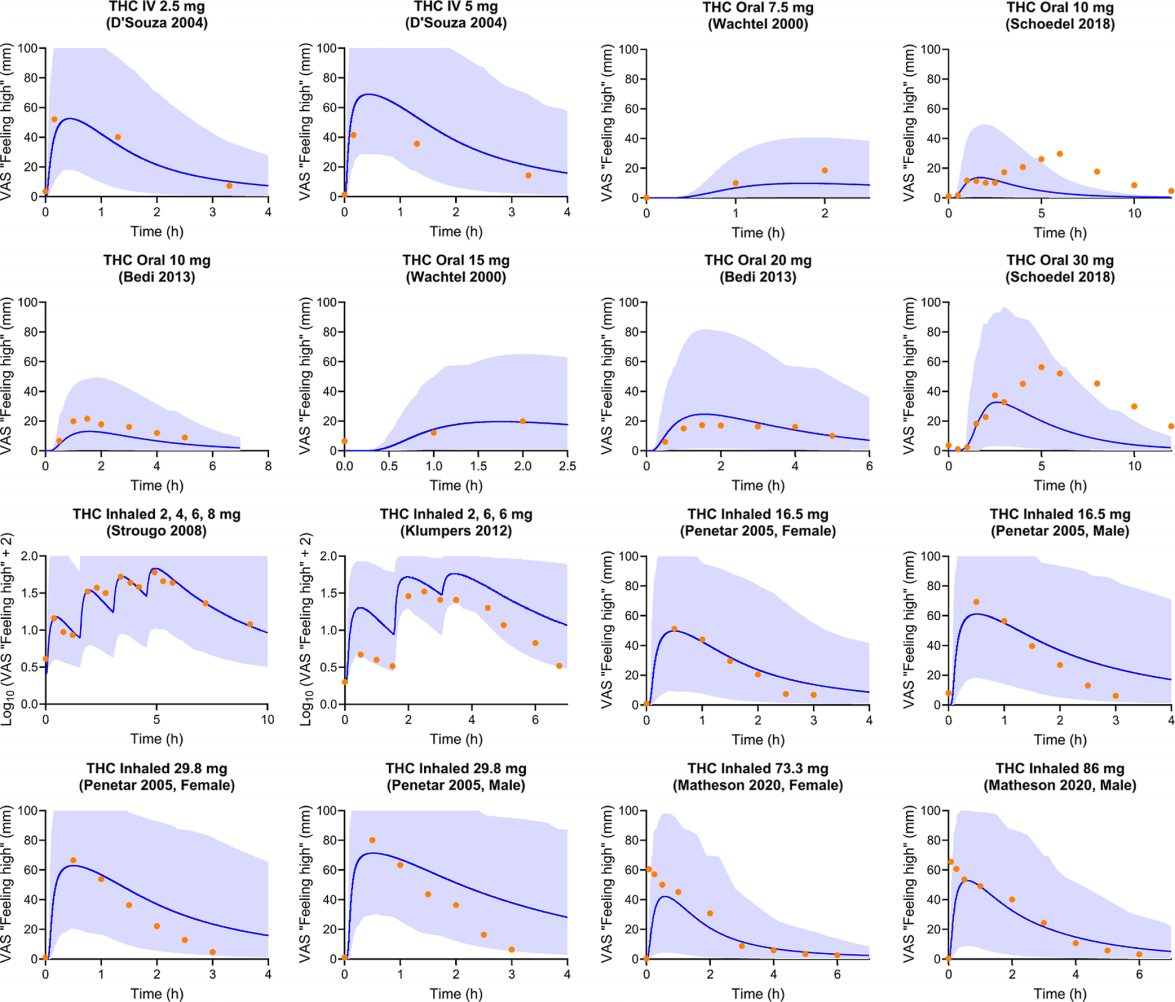
Observed vs PBPK–PD model-predicted VAS “feeling high”. The blue shaded areas represent the 5th to 95th percentiles of predicted values. The blue lines and orange circles represent the mean predicted value and observed VAS “feeling high” scores, respectively. Noted data for Strougo 2008 ^33^ and Klumpers 2012 ^44^ are presented as Log_10_(VAS + 2), as reported in the original studies.

The degree of feeling high following oral and inhaled THC administration at rest condition, ranging from 1 mg to 100 mg, were simulated. The dose–response relationship between THC dose and the degree of feeling high for both oral and inhaled administration is summarized in Figure 3. Our simulations show that the VAS “feeling high” scores increase with higher THC doses for both administration routes, with a sigmoidal relationship, particularly evident in the 5–50 mg range. For oral administration (Figure 3A), minimal effects are observed below 5 mg, with substantial increases occurring between 10 and 50 mg. For inhaled administration (Figure 3B), the dose–response curve shows similar characteristics but with greater potency at equivalent doses, as indicated by the vertical reference lines marking specific cannabis cigarette doses (2.55 mg, 12.75 mg, and 25.5 mg THC, equivalent to 1, 5, and 10 legal cannabis cigarettes, respectively).

**Figure 3:**
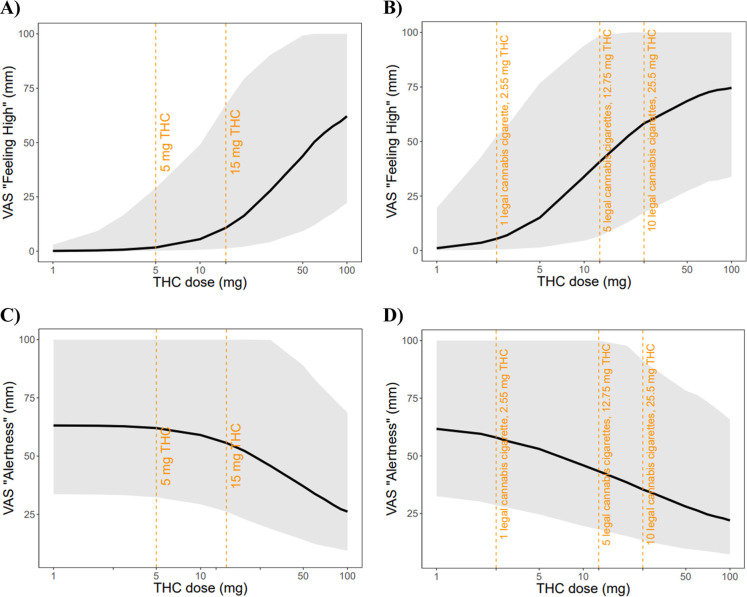
PBPK–PD model-predicted (A,B) VAS “feeling high” and (C,D) VAS “alertness” following (A,C) oral and (B,D) inhaled THC administration with a dose range from 1 mg to 100 mg at a rest condition. The gray shaded areas represent the 5th to 95th percentiles of predicted VAS scores. The black lines represent the mean predicted VAS scores. The orange dashed lines represent specific doses.

### 2.2 PBPK–PD Model for VAS “Alertness”

The PD model for VAS “alertness” was described using an indirect effect model, coded using the custom Lua models within the Simcyp. The absorption parameters for inhalation were from our prior studies (Table 1).^7,36^ Effect compartment models for THC and 11-OH-THC were tested. The results show that an effect compartment is unnecessary for the VAS “alertness” model. This may be because of the time lag caused by the indirect model part. A steady-state model for VAS “alertness” with zero-order input (*k*_in_) and first-order decline (*k*_out_) was established to describe the tonic aspects of the alertness when awakened. *E*_max,alertness_ and EC_50,alertness_ were assumed to be the same for THC and 11-OH-THC.^37,38^ A direct model was also tested but could not capture the relationship between THC exposure and the reduction in alertness well. The indirect response model used for alertness is particularly appropriate given the neurobiological mechanisms underlying THC’s effects on alertness. THC has been shown to modulate neurotransmitter systems critical for maintaining alertness, including noradrenergic, dopaminergic, and serotonergic pathways. The indirect model structure, with inhibition of the production (*k*_in_) by THC and 11-OH-THC, aligns with the physiological understanding that these compounds suppress the activity of alertness-promoting neural circuits rather than directly inducing sedation.

The developed PBPK–PD model captured the VAS “alertness” score well. Figure 1 compared the model-predicted trough VAS “alertness” against the observed.^33,50–52^ Optimized PD parameter values, including the maximum effect on VAS “alertness” (*E*_max,alertness_), the concentration required to obtain 50% of the maximum change of VAS “alertness” (EC_50,alertness_), and *k*_out_ are summarized in Table 1. The predicted peak VAS “alertness” score was within 1.3-fold of the observed peak in all trials included in the verification data sets (Figure 1). The ratio of predicted to observed mean trough of VAS “alertness” score (*R*_max,alertness_) ranged from 0.80 to 1.18. Additionally, all the observed VAS “alertness” scores fell within the fifth to 95th percentile of the predictions after THC administration (Figure 4). When predicting Strougo’s trial (2, 4, 6, and 8 mg THC inhaled), the mean VAS “alertness” score of the placebo group was used as the baseline of the THC treatment group.^33^ This is because the predose “alertness” score is smaller than the placebo group and the first two measurements postdose.

**Figure 4:**
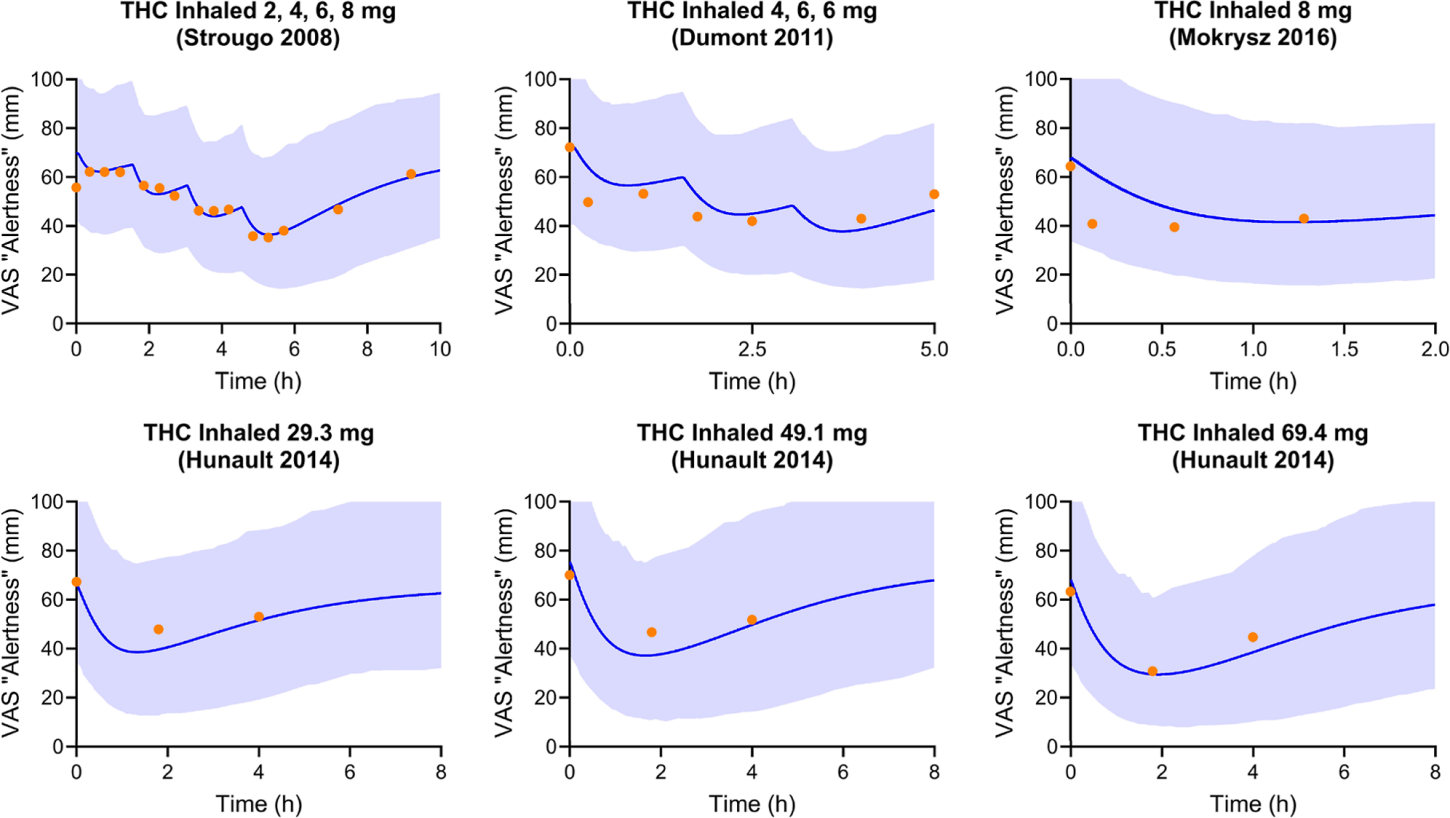
Observed vs PBPK–PD model-predicted VAS “alertness”. The blue shaded areas represent the 5th to 95th percentiles of predicted values. The blue lines and orange circles represent the mean predicted value and observed VAS “alertness” scores, respectively.

The reduction in alertness following oral and inhaled THC administration at rest condition, ranging from 1 mg to 100 mg, were simulated. The dose–response relationship between THC dose and reduction in alertness for both oral and inhaled administration is summarized in Figure 3. Interestingly, the alertness response shows a more gradual decline compared to the feeling high effect, with minimal reduction at lower doses (5 mg oral or 2.55 mg inhaled) and progressively greater effects at higher doses. The model predicts that substantial reductions in alertness occur primarily at doses above 15 mg for oral administration and above 12.75 mg for inhaled administration.

The route-dependent differences in potency captured by our model reflect the established pharmacokinetic (PK) differences between oral and inhaled administration, where inhaled THC bypasses first-pass metabolism and reaches the brain more rapidly.^53^ This explains the more rapid effects at equivalent doses for inhaled versus oral administration shown in our simulations. The wide, gray-shaded areas representing the fifth to 95th percentiles indicate considerable interindividual variability in both effects. This variability highlights the challenge of predicting individual responses and highlights the need for personalized dosing strategies in clinical applications. The reference doses marked by orange dashed lines provide clinically relevant benchmarks that could guide dosing decisions for both recreational users and medical cannabis patients. However, the simulations need to be interpreted with caution and require further data verification.

### 2.3 Limitations and Future Work

There are limitations to our study. First, the number of studies and available sample size for the VAS “feeling high” and “alertness” models were limited, which likely contributes to the observed high interindividual variability in the PD predictions. Second, a first-order absorption model was applied to oral and inhaled THC because of the limited understanding of the mechanisms for THC absorption. Third, our PBPK model captured THC exposure up to 86 mg. Whether the PK of THC remains linear beyond that dose is not known. Fourth, our model did not adjust the observed VAS “feeling high” and VAS “alertness” with placebo effects. The VAS scores are subjective measurements, which might be strongly impacted by the study designs (e.g., additional behavior measures and permitted activities during the study period such as watching movies) and could vary across individuals.^54^ It is difficult to differentiate THC effects from placebo effects. Finally, while our current PD models effectively link brain concentrations to subjective effects, they remain empirical in describing the direct PD action. Our models do not include the detailed mechanisms of how THC and 11-OH-THC interact with CB1 receptors or modulate neurotransmitter systems to ultimately produce the feeling high or a reduction in alertness.

In future studies, the predictive capability of our models could aid in dose optimization and therapeutic decision-making by providing insights into the dose–exposure–response relationship across different populations, including those with interindividual variability due to weight and age differences, or comorbidities. More mechanistic models, such as target binding models or quantitative systems pharmacology models, would offer a better understanding of THC’s exposure–response relationship. However, it is critical to recognize that the development of such sophisticated models will require further dedicated experimental studies to obtain the key parameters necessary for the model development and validation, including detailed receptor pharmacology data and quantitative measures of neurotransmitter dynamics *in vivo*. Furthermore, while our models focus on feeling high and alertness, these represent only two aspects of THC’s complex PD profile. Future work may incorporate additional cognitive, behavioral, or mood-related end points to provide a more comprehensive picture of THC’s effects. The limitations of VAS as a measurement tool should also be considered in future work. While VAS offers advantages in capturing subjective experiences on a continuous scale, it requires careful implementation and interpretation. Additionally, future studies should consider standardizing the administration and interpretation of VAS across research settings to improve comparability between studies. While differences in VAS scores between males and females have been observed in some studies, the underlying mechanisms for these differences, particularly whether they are sex-specific, remain unclear.^43,45^ Therefore, future studies should focus on comprehensively investigating sex differences in THC PD effects, ideally with larger, sex-stratified cohorts.

### 2.4 Conclusion

We developed and verified the first PBPK–PD models for VAS “feeling high” and VAS “alertness” based on THC and 11-OH-THC concentrations at the site of action using our previously published THC PBPK model. Our models accurately capture the observed VAS score changes across a broad dose range (2–86 mg) and multiple administration routes. The models show potential as predictive tools for optimizing THC dosing and guiding future studies. Our models establish a quantitative foundation that can be built upon to develop more sophisticated mechanistic models capable of exploring specific molecular interactions, receptor dynamics, and neurotransmitter system perturbations underlying THC’s diverse CNS impacts. As additional clinical data and mechanistic insights become available, our model can be expanded to incorporate further psychoactive and cognitive effects and to extrapolate predictions to specific populations, ultimately supporting more informed clinical decision-making.

## 3 Methods

### 3.1 PBPK–PD Software, Data Acquisition, and Parameter Assessment

PBPK–PD models were developed using the Simcyp PBPK Simulator (version 23, Certara, Sheffield, UK). The virtual healthy adult population was used in the model development. This virtual population was created by Certara, with parameters from public health databases, particularly the US National Health and Nutrition Examination Survey (NHANES) database; demographic data were obtained from real subjects who have taken part in phase I studies.^55,56^ Ten trials were simulated using study designs that closely matched the corresponding clinical trials, ensuring consistency in the VAS scores baseline, dosing regimen, number of subjects, age range, and proportion of females. All accessible clinical data, including THC concentration–time profiles and VAS “feeling high”–time profiles or VAS “alertness”–time profiles, were sourced from the published literature or digitized using WebPlotDigitizer (https://automeris.io/WebPlotDigitizer/).

### 3.2 Data Sets for Model Development

Inclusion criteria for THC clinical studies used for the PBPK–PD model development and verification were (1) studies involved healthy adult participants; (2) VAS “feeling high” or VAS “alertness” time profiles were reported; (3) THC dosing and/or concentration–time data were provided; and (4) THC was administered via the IV, oral, or inhalation routes. Exclusion criteria were (1) unspecified THC dose; and (2) reported a nonzero baseline THC concentration. In total, the observed data used to develop and verify the THC PBPK–PD model for VAS “feeling high” were derived from eight published clinical trials comprising 16 dosing regimens. The model for VAS “alertness” modeling was derived from four published clinical trials comprising six dosing regimens. Studies used for PBPK–PD model training and verification are presented in Table S1.

### 3.3 PBPK Model Development

A PBPK model of THC, previously published by our group, was developed using the Simcyp Simulator (version 22). Details of the PBPK model inputs for THC and the sensitivity parameters for THC exposure have been previously described.^7^ The model included IV, oral, and inhaled THC administrations. The disposition of both THC and 11-OH-THC was characterized using a whole-body PBPK model with predicted tissue partition coefficients, as illustrated in Figure S1. Tissue distribution, including the brain, was assumed to be perfusion limited. The workflow for the model development is summarized in Figure 5.

**Figure 5:**
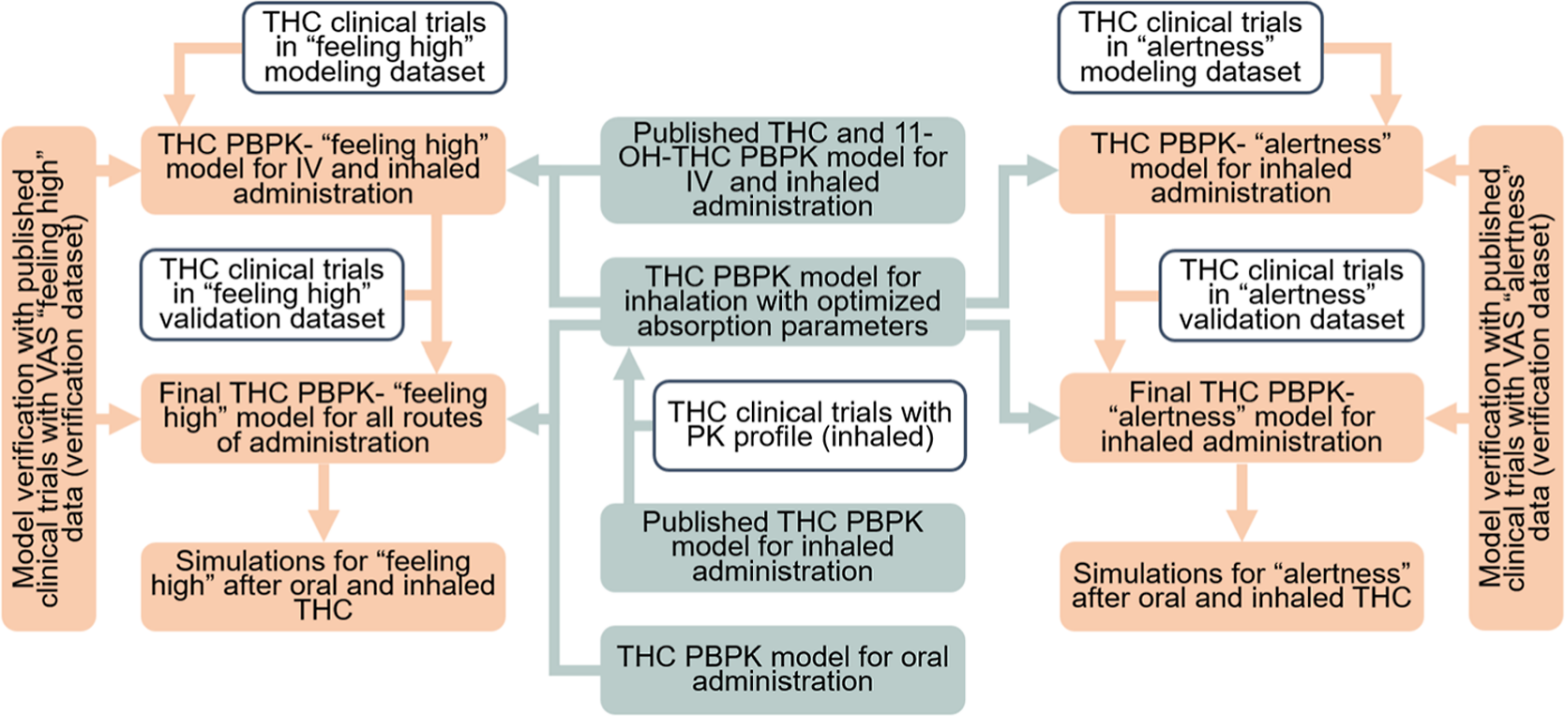
General workflow for Δ-9-tetrahydrocannabinol (THC) physiologically based pharmacokinetic–pharmacodynamic (PBPK–PD) model for visual analogue scale (VAS) “feeling high” and VAS “alertness”. PK, pharmacokinetic; IV, intravenous.

The IV and oral clinical trials included in the study did not provide concentration–time profiles for THC. Therefore, our previously developed PBPK models for IV and oral THC were used without modification.^7,36^ For inhaled THC trials, there were two types of PK data: (type 1) clinical trials provided THC concentration–time profiles and included in our previous THC PBPK and PBPK–PD study; (type 2) clinical trials reported only doses and no THC concentration–time profiles available. For type 1 data, all parameters are from the prior PBPK model. For type 2 data, the Lung *f*_a_ and first-order absorption rate constant for lung (Lung *k*_a_) values from prior studies were tested, and values that achieve the closest peak or trough VAS scores would be used. All the other PBPK parameters are from prior models.^7,36^

### 3.4 PBPK–PD Model Development

Direct models with or without an effect compartment were tested. Indirect models were not tested because (1) feeling high score baselines were zero, where a steady-state model cannot be established; and (2) the mechanism of feeling high is not clear, as the type of effect (inhibition/activation on input/output) on feeling high is unknown. The VAS “feeling high” model driven by THC and 11-OH-THC was established and verified using a nonlinear maximum effect (*E*_max_) model with an effect compartment (eqs 1–3)­

1 $$\frac{dC_{THC,effect}}{dt}=k_{e0,THC}\times \left(C_{THC,brain}-C_{THC,effect}\right)$$

2 $$\frac{dC_{11‐PH‐THC,effect}}{dt}=k_{e0,11‐OH‐THC}\times \left(C_{11‐OH‐THC,brain}-C_{11‐OH‐THC,effect}\right)$$

3 $$“Feeling\;high”\;\left(mm\right)=E_{\max,high}\times \frac{{\left(C_{THC,effect}+C_{11‐OH‐THC,effect}\right)}^{Hill1}}{{{EC}_{50,high}}^{Hill1}+{\left(C_{THC,effect}+C_{11‐OH‐THC,effect}\right)}^{Hill1}}$$

where *C*_THC,effect_ is the total THC concentration in the effect compartment linked to the brain compartment; *k*_e0,THC_ is the THC effect compartment transportation rate constant, *k*_1e_ is same as *k*_e0_ for both THC and 11-OH-THC; *C*_THC,brain_ is the total THC concentration in the brain compartment; *C*_11‑OH‑THC,effect_ is the total 11-OH-THC concentration in the effect compartment associated with the brain; *k*_e0,11‑OH‑THC_ is the 11-OH-THC effect compartment transportation rate constant; *C*_11‑OH‑THC,brain_ is the total 11-OH-THC concentration in the brain compartment; “Feeling high” (mm) is the VAS “feeling high” score; *E*_max,high_ is the maximum increase fraction of THC and 11-OH-THC on VAS “feeling high”; Hill1 is the power parameter for VAS “feeling high” model; EC_50,high_ is the half maximal effective concentration of THC and 11-OH-THC in the effect compartment. The EC_50,high_ and *E*_max,high_ of THC and 11-OH-THC were assumed to be the same.^37,38^ The *k*_e0_, *E*_max,high_, and EC_50,high_ were estimated using the Simcyp Parameter Estimation module and optimized manually with clinical data from the modeling data set to best capture the VAS “feeling high” score changes induced by THC and 11-OH-THC. Interindividual variability of *E*_max_ from a popPK/PD model was applied to our VAS “feeling high” model. Interindividual variability for EC_50,high_ was reduced from the reference value of 126% to 70% to prevent the occurrence of nonphysiological (negative) EC_50,high_ values during simulations.^33^

Direct model and indirect models, with or without effect compartment, were tested for the VAS “alertness”. The THC-induced input inhibition of alertness was assumed to be driven by the sum of the total THC and 11-OH-THC concentrations in the brain compartment and was described by an *E*_max_ model (eqs 4–6).

4 $$\frac{d\left(VAS\;“alertness”\right)}{dt}=k_{in}\times \left(1-E_{THC,alertness}\right)-k_{out}\times \left(VAS\;“alertness”\right)$$

5 $$k_{in}=Baseline\times k_{out}$$

6 $$E_{THC,alertness}=E_{\max,alertness}\times \frac{{\left(C_{THC,brain}+C_{11‐OH‐THC,brain}\right)}^{Hill2}}{{{EC}_{50,alertness}}^{Hill2}+{\left(C_{THC,brain}+C_{11‐OH‐THC,brain}\right)}^{Hill2}}$$

where *k*_in_ is the zero-order input constant for VAS “alertness”; Baseline is the baseline of VAS “alertness” before THC dosing; *E*_THC,alertness_ is the fraction of THC and 11-OH-THC effect on decreasing alertness input; *k*_out_ is the first-order decline constant of VAS “alertness”; *E*_max,alertness_ is the maximum decrease fraction of THC and 11-OH-THC on zero-order alertness input; Hill2 is the power parameter for the VAS “alertness” model; EC_50,alertness_ is the half maximal effective concentration of THC and 11-OH-THC in the brain compartment. The parameter estimation and optimization methods are the same as for the feeling high model. Interindividual variabilities of *E*_max_ and EC_50_ were set to 30% as there were no reference values.

Our PD models were written with custom differential equations; the formulations above could be reproduced in other PBPK software that can predict the brain concentrations of THC and supports custom models, including the open-source PBPK simulator PK-Sim.

### 3.5 Model Verification

Model verification was conducted using clinical trials with the THC administration. The workflow is shown in Figure 5. For all studies, prediction performance was assessed by determining whether the observed VAS scores fell within the fifth to 95th percentiles of the predicted values. For the VAS “feeling high” clinical trials that provided PK profiles and those without PK profiles but with multiple dosing regimens within the same trial, prediction performance was evaluated by checking whether the predicted peak VAS “feeling high” score fell within a 2-fold range of the observed values.^57^ For VAS “alertness” trials, a 1.3-fold range was applied. Although the 2-fold range is more commonly used in PBPK model predictions, the 1.3-fold range, which has been used in some studies, was chosen for VAS “alertness” to better reflect the baseline distribution in these trials.^57^

### 3.6 Simulation

The degree of feeling high and reduction in alertness caused by THC were simulated using the verified THC PBPK–PD model. THC administered orally or by inhalation ranging from 1 mg to 100 mg were simulated with the “Sim-Healthy Volunteers” virtual population. The therapeutic doses of oral THC with two doses (5 mg or 15 mg), and inhaled THC corresponding to three doses of legal cannabis cigarettes (1, 5, or 10 cigarettes, assuming a cigarette weight of 850 mg and a THC concentration of 0.3%, equating to 2.55 mg THC per cigarette^58^), were also simulated. In each simulation, 20 trials following a single THC dose were simulated with 25 healthy adults between the ages of 18 and 65. The baseline for feeling high was set at 0 mm because subjects experience no high sensation prior to THC administration, whereas the baseline for alertness was set at 60 mm, which is consistent with the baseline reported in clinical trials. The sex ratio was set at 1:1 for the simulation.

## Supplementary Material



## Notes

Figure S1 schematic diagram of the organ compartments used in the whole-body PBPK model for THC and 11-OH-THC; Table S1 PBPK–PD predicted and observed VAS “feeling high” and VAS “alertness” scores following THC administration in healthy adults (PDF)

## Abbreviations

U.S.: United States
THC: Δ-9-tetrahydrocannabinol
CB1: cannabinoid receptor 1
VAS: visual analogue scale
CNS: central nervous system
NE: norepinephrine
DA: dopamine
5-HT: 5-hydroxytryptamine
LC–NE: locus coeruleus–norepinephrine
popPK/PD: population pharmacokinetics/pharmacodynamics
11-OH-THC: 11-hydroxy-THC
PBPK: physiologically based pharmacokinetic
PBPK–PD: physiologically based pharmacokinetic–pharmacodynamics
PD: pharmacodynamic
*E*_max,high_: maximum effect on VAS “feeling high”
EC_50,high_: concentration required to obtain 50% of the maximum change of VAS “feeling high”
IV: intravenous
*k*_e0_: transport rate constants for the effect compartment
*R*_max,feeling high_: ratio of predicted to observed mean peak VAS “feeling high” score
*t*_max_: time to peak plasma concentration
EHC: enterohepatic circulation
*k*_in_: zero-order input constant of the alertness compartment
*k*_out_: first-order decline constant of the alertness compartment
*E*_max,alertness_: maximum effect on VAS “alertness”
EC_50,alertness_: concentration required to obtain 50% of the maximum change of VAS “alertness”
*R*_max,alertness_: ratio of predicted to observed mean trough of VAS “alertness” score
PK: pharmacokinetics
NHANES: National Health and Nutrition Examination Survey
Lung *f*_a_: fraction of the drug absorbed by the lung
Lung *k*_a_: first-order absorption rate constant for lung
*E*_max_: maximum effect
