# Supplementary material for: Predicting Δ‑9-Tetrahydrocannabinol-Induced Psychoactive and Cognitive Effects: A PBPK–PD Approach to Quantifying Feeling High and Reduced Alertness
Source: ACS Chem Neurosci. 2025 Jul 22;16(15):3059–69. doi: 10.1021/acschemneuro.5c00417 (PMC12333010; doi:10.1021/acschemneuro.5c00417)
Supplement: Supplementary file 1 [file cn5c00417_si_001.pdf]

SUPPLEMENTARY MATERIALS

Predicting Δ-9-tetrahydrocannabinol-Induced Psychoactive and Cognitive Effects: A PBPK-PD Approach to Quantifying Feeling High and Reduced Alertness

*Lixuan Qian, Zhu Zhou**

Department of Chemistry, York College, City University of New York, Jamaica, NY 11451, USA

*Correspondence:

Zhu Zhou, Ph.D., Email: zzhou1@york.cuny.edu.

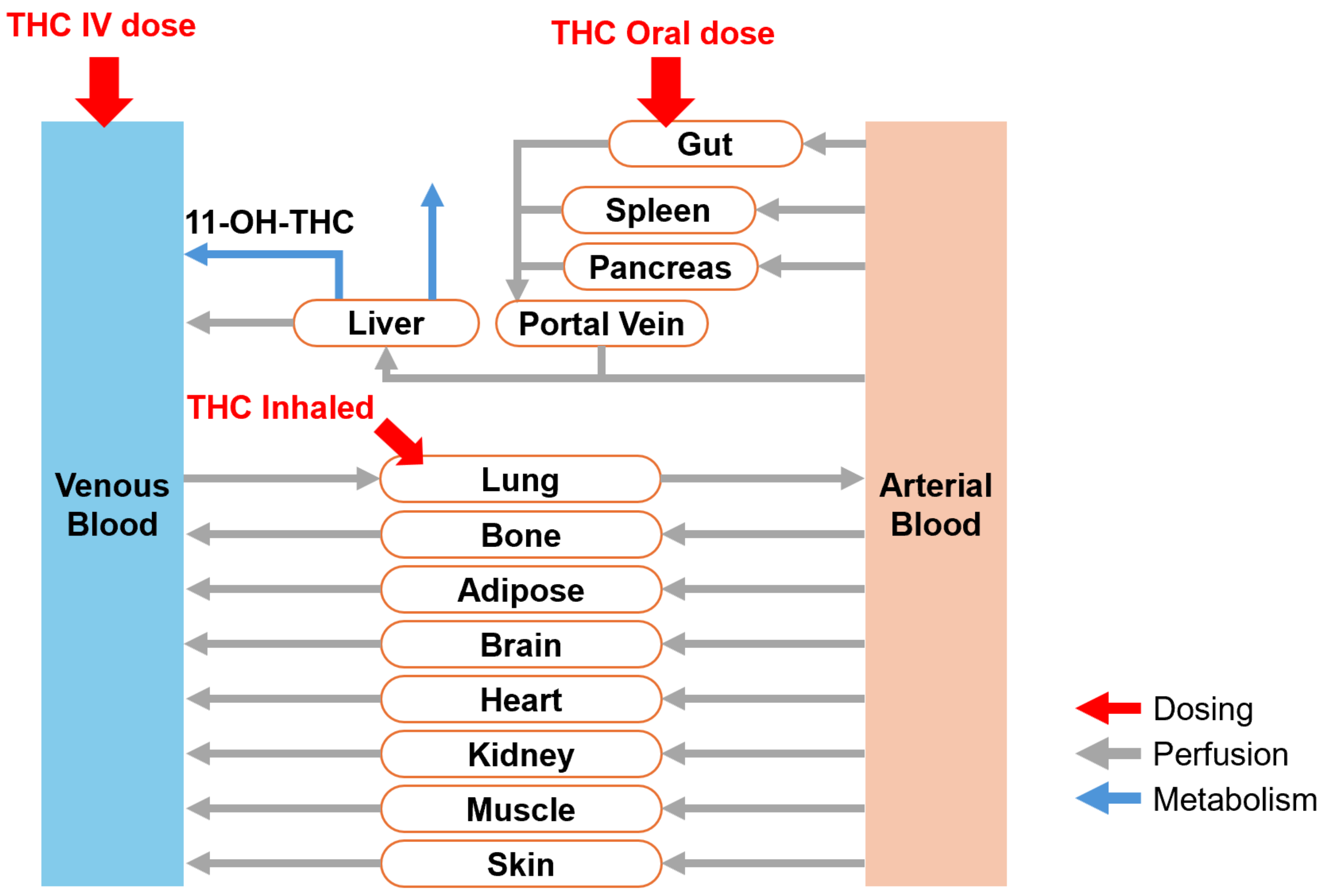

Figure S1. Schematic diagram of the organ compartments used in the whole-body physiologically based pharmacokinetic model for Δ-9-tetrahydrocannabinol (THC) and 11-hydroxy-THC (11-OH-THC). 11-OH-THC is metabolized from THC in the liver and then enters the systemic circulation. IV, intravenous.

Table S1. PBPK-PD predicted and observed VAS "feeling high" and VAS "alertness " scores following delta-9-tetrahydrocannabinol (THC) administration in healthy adults.

Formulation	Trial	PD Group [a]	PK Group [b]	Dose regimen	Absorption Parameters	Observed R_{max} (mm)	Predicted R_{max} (mm)	R_{max} Predicted: Observed
**VAS "feeling high"**								
IV	D'Souza 2004[1]	Modeling	/	2.5 mg	/	52.1	52.6	1.01
		Modeling	/	5 mg	/	41.5	59.6	1.44
Oral	Wachtel 2000[2]	Verification	/	7.5 mg	$f_a = 0.45$, $k_a = 0.7$/h	18.5	9.8	0.53
		Verification	/	15 mg	$f_a = 0.45$, $k_a = 0.7$/h	19.9	19.6	0.98
	Bedi 2013[3]	Verification	/	10 mg	$f_a = 0.45$, $k_a = 0.7$/h	21.6	13.1	0.61
		Verification	/	20 mg	$f_a = 0.45$, $k_a = 0.7$/h	17.3	24.7	1.43
	Schoedel 2018[4]	Verification	/	10 mg	$f_a = 0.45$, $k_a = 0.7$/h	29.7	13.7	0.46
		Verification	/	30 mg	$f_a = 0.45$, $k_a = 0.7$/h	56.3	32.0	0.57
Inhaled	Strougo 2008[5]	Modeling	Type 1	2, 4, 6, 8 mg	lung $f_a = 0.6$, lung $k_a = 200$/h	1.78 [c]	1.83 [c]	1.03
	Matheson 2020[6]	Verification	Type 1	73.3 mg [d]	lung $f_a = 0.025$, lung $k_a = 12$/h	60.5	42.2	0.70
		Verification	Type 1	86 mg [e]	lung $f_a = 0.05$, lung $k_a = 12$/h	65.5	52.9	0.81
	Penetar 2005[7]	Verification	Type 2	16.5 mg [d]	lung $f_a = 0.22$, lung $k_a = 200$/h	51.3	49.9	0.97
		Verification	Type 2	16.5 mg [e]	lung $f_a = 0.4$, lung $k_a = 200$/h	69.3	62.9	0.91
		Verification	Type 2	29.8 mg [d]	lung $f_a = 0.22$, lung $k_a = 200$/h	66.5	62.9	0.95
		Verification	Type 2	29.8 mg [e]	lung $f_a = 0.4$, lung $k_a = 200$/h	80.1	71.4	0.89
	Klumpers 2012[8]	Verification	Type 2	2, 6, 6 mg	lung $f_a = 0.6$, lung $k_a = 200$/h	1.52 [c]	1.76 [c]	1.16
**VAS "alertness"**								
Inhaled	Strougo 2008[5]	Modeling	Type 1	2, 4, 6, 8 mg	lung $f_a = 0.6$, lung $k_a = 200$/h	35.3	36.4	1.03
	Hunault 2014[9]	Verification	Type 1	29.3 mg	lung $f_a = 0.22$, lung $k_a = 12$/h	47.9	39.6	0.83
		Verification	Type 1	49.1 mg	lung $f_a = 0.22$, lung $k_a = 12$/h	46.7	37.3	0.80
		Verification	Type 1	69.4 mg	lung $f_a = 0.22$, lung $k_a = 12$/h	30.8	29.5	0.96
	Dumont 2011[10]	Verification	Type 2	4, 6, 6 mg	lung $f_a = 0.6$, lung $k_a = 200$/h	42.0	45.1	1.07
	Mokrysz 2016[11]	Verification	Type 2	8 mg	lung $f_a = 0.9$, lung $k_a = 200$/h	39.5	46.7	1.18

R_{max}, mean peak VAS "feeling high" score or mean trough VAS "alertness" score; IV, intravenous; /, not applied; f_a, fraction of oral absorption; k_a, first-order oral absorption rate constant; lung f_a, fraction of drug absorbed from the lung; lung k_a, first-order absorption rate constant of lung absorption.

[a] Modeling: dataset was used for developing the PD model; Verification: dataset was used for PD model verification.

[b] Type 1: clinical trials with THC concentration-time profiles included in our previous THC PBPK study and PBPK-PD study[12, 13]; Type 2: clinical trials provided only dosing information but no THC concentration-time profiles.

[c] The Log_{10}(VAS + 2) was reported in the original studies.

[d] female subjects only.

[e] male subjects only.

REFERENCES

(1) D'Souza, D. C.; Perry, E.; MacDougall, L.; Ammerman, Y.; Cooper, T.; Wu, Y. T.; Braley, G.; Gueorguieva, R.; Krystal, J. H. The psychotomimetic effects of intravenous delta-9-tetrahydrocannabinol in healthy individuals: implications for psychosis. Neuropsychopharmacology 2004, 29 (8), 1558-1572. DOI: 10.1038/sj.npp.1300496

(2) Wachtel, S. R.; de Wit, H. Naltrexone does not block the subjective effects of oral Delta(9)-tetrahydrocannabinol in humans. Drug Alcohol Depend 2000, 59 (3), 251-260. DOI: 10.1016/s0376-8716(99)00127-1

(3) Bedi, G.; Cooper, Z. D.; Haney, M. Subjective, cognitive and cardiovascular dose-effect profile of nabilone and dronabinol in marijuana smokers. Addict Biol 2013, 18 (5), 872-881. DOI: 10.1111/j.1369-1600.2011.00427.x

(4) Schoedel, K. A.; Szeto, I.; Setnik, B.; Sellers, E. M.; Levy-Cooperman, N.; Mills, C.; Etges, T.; Sommerville, K. Abuse potential assessment of cannabidiol (CBD) in recreational polydrug users: A randomized, double-blind, controlled trial. Epilepsy Behav 2018, 88, 162-171. DOI: 10.1016/j.yebeh.2018.07.027

(5) Strougo, A.; Zuurman, L.; Roy, C.; Pinquier, J. L.; van Gerven, J. M.; Cohen, A. F.; Schoemaker, R. C. Modelling of the concentration--effect relationship of THC on central nervous system parameters and heart rate -- insight into its mechanisms of action and a tool for clinical research and development of cannabinoids. J Psychopharmacol 2008, 22 (7), 717-726. DOI: 10.1177/0269881108089870

(6) Matheson, J.; Sproule, B.; Di Ciano, P.; Fares, A.; Le Foll, B.; Mann, R. E.; Brands, B. Sex differences in the acute effects of smoked cannabis: evidence from a human laboratory study of young adults. Psychopharmacology (Berl) 2020, 237 (2), 305-316. DOI: 10.1007/s00213-019-05369-y

(7) Penetar, D. M.; Kouri, E. M.; Gross, M. M.; McCarthy, E. M.; Rhee, C. K.; Peters, E. N.; Lukas, S. E. Transdermal nicotine alters some of marihuana's effects in male and female volunteers. Drug Alcohol Depend 2005, 79 (2), 211-223. DOI: 10.1016/j.drugalcdep.2005.01.008

(8) Klumpers, L. E.; Cole, D. M.; Khalili-Mahani, N.; Soeter, R. P.; Te Beek, E. T.; Rombouts, S. A.; van Gerven, J. M. Manipulating brain connectivity with delta(9)-tetrahydrocannabinol: a pharmacological resting state FMRI study. Neuroimage 2012, 63 (3), 1701-1711. DOI: 10.1016/j.neuroimage.2012.07.051

(9) Hunault, C. C.; Bocker, K. B.; Stellato, R. K.; Kenemans, J. L.; de Vries, I.; Meulenbelt, J. Acute subjective effects after smoking joints containing up to 69 mg Delta9-tetrahydrocannabinol in recreational users: a randomized, crossover clinical trial. Psychopharmacology (Berl) 2014, 231 (24), 4723-4733. DOI: 10.1007/s00213-014-3630-2

(10) Dumont, G. J.; van Hasselt, J. G.; de Kam, M.; van Gerven, J. M.; Touw, D. J.; Buitelaar, J. K.; Verkes, R. J. Acute psychomotor, memory and subjective effects of MDMA and THC co-administration over time in healthy volunteers. J Psychopharmacol 2011, 25 (4), 478-489. DOI: 10.1177/0269881110376687

(11) Mokrysz, C.; Freeman, T. P.; Korkki, S.; Griffiths, K.; Curran, H. V. Are adolescents more vulnerable to the harmful effects of cannabis than adults? A placebo-controlled study in human males. Transl Psychiatry 2016, 6 (11), e961. DOI: 10.1038/tp.2016.225

(12) Qian, L.; Zhang, T.; Dinh, J.; Paine, M. F.; Zhou, Z. Physiologically Based Pharmacokinetic Modeling of Cannabidiol, Delta-9-Tetrahydrocannabinol, and Their Metabolites in Healthy Adults After Administration by Multiple Routes. Clin Transl Sci 2025, 18 (1), e70119. DOI: 10.1111/cts.70119

(13) Qian, L.; Zhou, Z. Quantifying Heart Rate Changes After Delta-9-Tetrahydrocannabinol Administration Using a PBPK-PD Model in Healthy Adults. Pharmaceutics 2025, 17 (2). DOI: 10.3390/pharmaceutics17020237
